# Perforation of gastric volvulus within a giant hiatus hernia secondary to alendronate: A rare complication of bisphosphonate use^☆^

**DOI:** 10.1016/j.ijscr.2020.06.068

**Published:** 2020-06-20

**Authors:** Bianca Kwan, Jason Wong

**Affiliations:** Department of Surgery, Redcliffe Hospital, Anzac Avenue, Redcliffe, QLD, Australia

**Keywords:** Alendronate, Bisphosphonates, Massive hiatus hernia, Perforated gastric volvulus, Case report

## Abstract

**Introduction:**

Bisphosphonates are commonly used in the treatment of metabolic bone disease. However, they are associated with gastrointestinal side effects including acid reflux, mucosal erosion, and oesophageal stricture. We present a rare case of alendronate causing perforated gastric volvulus in a patient with giant hiatus hernia.

**Presentation of case:**

An 82-year-old woman presented to our hospital with central chest pain, palpitations and new onset atrial fibrillation on the background of 2 weeks of vomiting. Computed tomography (CT) imaging revealed a perforated, mixed type organoaxial/mesoenteroaxial gastric volvulus within a giant hiatus hernia, with partial gastric outlet obstruction. The patient underwent laparoscopic reduction of hiatus hernia and gastric volvulus, conversion to laparotomy, and distal gastrectomy with Bilroth II reconstruction. An alendronate tablet was found in the right mediastinum. The patient had a prolonged post-operative course and was discharged home after completing extensive physical rehabilitation.

**Discussion:**

Bisphosphonates are widely used to treat metabolic bone disease, however can have devastating adverse effects on the gastrointestinal tract. There are a number of mechanisms postulated for how these medications cause injury to the gastric and oesophageal mucosa.

**Conclusion:**

This case illustrates the importance of considering the gastrointestinal effects associated with bisphosphonates when prescribing them to patients, especially those with functional or anatomical disorders of the gastrointestinal tract. The presence of a large hiatus hernia should be a contraindication to prescribing alendronate.

## Highlights

• A case of perforation due to alendronate in patient with gastric volvulus within giant hiatus hernia.
• Bisphosphonates can have devastating gastrointestinal side effects.
• Gastric or oesophageal perforation due to bisphosphonates is rare but serious.
• Bisphosphonates should be avoided in those with functional or anatomical disorders of the gastrointestinal tract.

## 1 Introduction

Bisphosphonates are commonly used in the treatment of metabolic bone disease such as osteoporosis and Paget’s disease. Bisphosphonates decrease bone resorption by reducing activity of osteoclasts, the cells responsible for breakdown of bone matrix [2].

These medications are also associated with a number of adverse events such as osteonecrosis of the jaw and atypical femoral fractures. However, by far the most common negative consequences of bisphosphonates are gastrointestinal side effects, including acid reflux, mucosal erosion, and oesophageal stricture [1]. In those unable to tolerate these gastrointestinal effects associated with oral bisphosphonates, intravenous preparations exist.

We present a rare case of perforated gastric volvulus in a patient with massive hiatus hernia, where the perforation is likely secondary to alendronate. This is the only reported case in the literature. This case has been reported in line with the SCARE criteria [2].

## 2 Presentation of case

### 2.1 Case history

An 82-year-old woman presented to our hospital with central chest pain, palpitations and new onset atrial fibrillation with rapid ventricular rate. This was on the background of 2 weeks of vomiting and 6 kg associated weight loss. The patient had a past history of osteoporosis, angina, hypertension and osteoarthritis.

Laboratory investigations revealed an elevated white cell count of 27.3 × 10^9^/L, with a neutrophilia of 25.76 × 10^9^/L. Haemoglobin was 13.2 g/dL. The patient had an acute kidney injury with creatinine of 155 μmol/L (baseline 60 μmol/L). Venous blood gas showed a normal pH of 7.33, with an elevated lactate of 4.1 mmol/L.

**Fig. 1:**
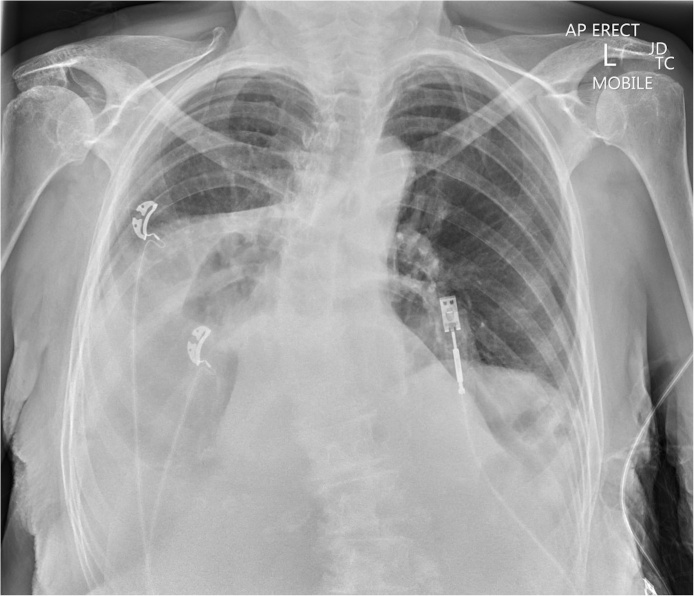
Chest radiograph showing a large retrocardiac shadow with an intrathoracic gas bubble on the right, indicating a large hiatus hernia.

**Fig. 2:**
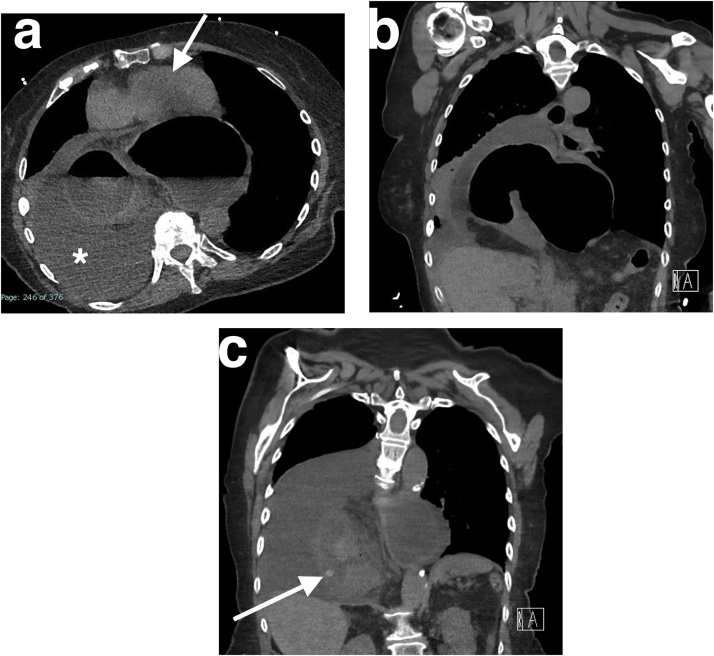
Computed tomography images of the chest, showing gastric volvulus within a giant hiatal hernia. (a); Axial image showing associated right pleural effusion (white asterisk) and compression of the heart (white arrow) and right lower lobe. (b); Coronal image showing the entire stomach within the thoracic cavity. (c); A tablet (white arrow) can be seen in the right mediastinum on this coronal image.

Chest X-ray showed evidence of a large hiatus hernia (Fig. 1). Subsequent contrast computed tomography (CT) imaging of the chest and abdomen revealed a mixed organoaxial/mesoenteroaxial gastric volvulus within a large hiatus hernia. The entire stomach was within the thoracic cavity. There was a degree of outlet obstruction but no gastric torsion. An associated large right pleural effusion with compressive atelectasis of the right lower lobe could be seen. There was subsequent compression of the heart against the sternum. A small hyperdensity could be seen in the right mediastinum, thought to be a tablet (Fig. 2a, b, c).

### 2.2 Operative techniques

**Fig. 3:**
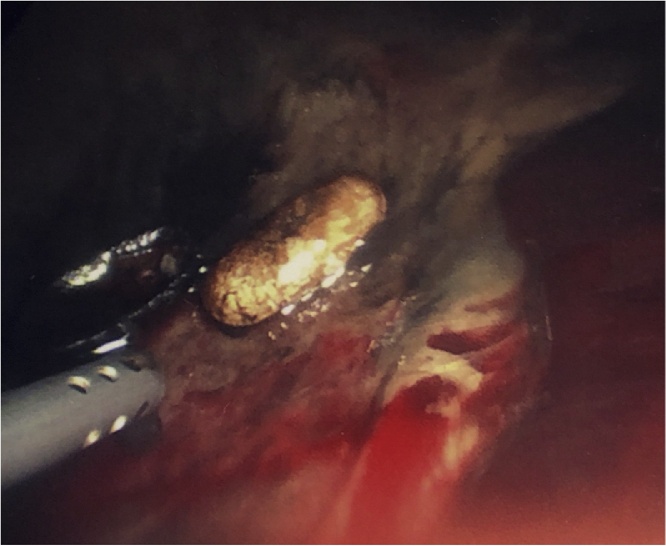
Intraoperative photograph of white oval tablet, likely Alendronate, found in the right mediastinum.

Laparoscopy was performed where contamination of all quadrants of the abdomen was found. There was a large hiatal hernia, with the entire stomach and first part of the duodenum located within the mediastinum. There was gross contamination of the mediastinum with mediastinitis present, along with food matter and a single medication tablet (Fig. 3). Lavage and laparoscopic reduction of the hiatus hernia and gastric volvulus was performed, with reduction and excision of hernia sac. When the source of perforation was unable to be found, the procedure was converted to exploratory laparotomy and a 1.2 cm perforation was found in the gastric antrum. The aforementioned white, oval-shaped medication tablet was found in the right mediastinum, adjacent to the site of perforation.

Distal gastrectomy with Bilroth II reconstruction were performed, with suture closure of the hiatus with 0-Novafil (Covidien, Dublin, Ireland). A surgical drain was placed through the hiatus into the mediastinum. Two further drains were placed in the upper abdomen and pelvis.

Postoperatively, review of the patient’s regular medications with the assistance of the hospital pharmacist revealed 70 mg of Alendronate in combination with calcium and vitamin D, taken once weekly. The bisphosphonate was the only white, oval-shaped tablet out of all the patient’s regular medications. The patient was not taking non-steroidal anti-inflammatory drugs (NSAIDs) or any other gastropathic medications.

The patient had a prolonged admission due to delirium, postoperative ileus, and reluctance to engage with allied health services. This resulted in severe physical deconditioning and poor nutrition. She was stepped down to a rehabilitation facility on the 41st postoperative day. After extensive physical rehabilitation for a further 27 days, the patient was eventually discharged home independently. She has subsequently been non-compliant with any follow-up appointments offered to her at our hospital.

## 3 Discussion

The authors postulate the alendronate tablet found intraoperatively could have caused, or at least contributed to, perforation of this patient’s gastric volvulus. While this association cannot be proven, we feel it is certainly possible, due to the proximity of the tablet to the perforation site at operation, and the known erosive effects bisphosphonates can have on the gastrointestinal mucosa. Stasis of the alendronate tablet secondary to volvulus with partial gastric outlet obstruction would have led to mucosal ulceration and subsequent perforation.

Gastrointestinal adverse effects are well-established with oral nitrogen-containing bisphosphonates such as alendronate and risedronate. They can commonly cause abdominal pain and discomfort, dyspepsia, oesophagitis/gastritis, heartburn, nausea and gastro-oesophageal reflux [3,4]. There is mixed evidence on whether there is any difference in tolerability between risedronate and alendronate [5,6], with some studies suggesting lower rates of adverse events with risedronate and others finding no difference [4,7].

Bisphosphonates can cause adverse effects from damage to the both oesophageal or gastric mucosa. Oesophageal injury is more commonly studied and described. At endoscopy, oesophageal irritation, ulceration and stricture can be seen. A classic endoscopic and pathological appearance has even been characterised in studies of patients taking alendronate. Thick, white exudate loosely adherent to circumscribed white erosions is seen endoscopically. Pathological examination of this exudate reveals birefringent, crystalline material resembling the matrix material of alendronate [8,9].

In addition to oesophageal injury, evidence also suggests gastric and duodenal irritation secondary to the use of oral bisphosphonates. Endoscopic studies have shown alendronate causes gastric erosions and ulcers within as little as four days after initiating treatment [10,11]. There is also likely a synergistic ulcerogenic effect when used with NSAIDs [12]. This is important in the elderly population likely to be using bisphosphonates for osteoporosis, as they are also commonly using NSAIDs for chronic pain from conditions such as arthritis.

To avoid gastrointestinal irritation, manufacturers recommend that patients take the medication with a full glass of water and remain upright for at least 30 min after administration and until after the first meal of the day. There has been much evidence to show that bisphosphonates cause oesophageal irritation. Some postulate this is due to incorrect administration of the drug and failure of patients to follow the aforementioned dosing instructions. However, studies have shown some a significant proportion of patients still developed gastrointestinal side effects despite compliance with administration instructions [10].

The exact biochemical mechanism of mucosal injury from bisphosphonates is still being investigated. It is thought that in a mechanism similar to that of NSAIDs, bisphosphonates cause gastric mucosal injury by inducing cellular injury, cellular lipid peroxidation, and superoxide production [13]. Other processes, such as the involvement of specific T cell populations and interference with the mevalonate pathway also appear to be involved [10].

Pill oesophagitis, which is the prolonged exposure of the mucosa to the caustic alendronate tablet, has been postulated as a mechanism for oesophageal irritation and perforation [14]. A topical irritant effect of oral bisphosphonates on the gastric mucosa has also been shown, due to reduction in the protective hydrophobic barrier [15,16]. This is supported by the finding that most alendronate gastric ulcers are found in the greater curve of the gastric antrum [3], where tablets would usually settle due to gravity [10]. In addition, the low pH of gastric contents causes alendronate to be converted to its free acid form, which has been shown to damage oesophageal mucosa. This conversion of the drug in those with gastro-oesophageal reflux or hiatus hernia could lead to increased risk of mucosal damage [10].

The white, oval-shaped tablet found intraoperatively in this case was likely the patient’s regular Alendronate. We believe this medication was unable to be cleared from the patient’s stomach due to her gastric volvulus and massive hiatus hernia. This sustained mucosal contact then caused a perforated gastric ulcer due to the erosive properties of the medication.

The gastrointestinal adverse effects of bisphosphonates should be carefully considered when prescribing this medication to patients. Presence of oesophageal strictures, Barrett’s oesophagus and ulcers in the upper gastrointestinal tract are already contraindications to their use. Due to their erosive properties, we believe they should also be avoided in those with functional or anatomical disorders of the upper gastrointestinal tract, such as massive hiatus hernia, oesophageal diverticulum, gastroparesis and achalasia.

### 3.1 Conclusion

This is the only reported case in the literature where alendronate is postulated to be the cause of perforation in a gastric volvulus with massive hiatus hernia. Although rare, oesophageal and gastric perforations are devastating complications secondary to oral bisphosphonates. This case highlights how they should be avoided in those with functional or anatomical disorders of the gastrointestinal tract, such as massive hiatus hernia.

## Declaration of Competing Interest

No conflict of interest exists.

## Sources of funding

No sources of funding.

## Ethical approval

We have reported a single case, not a clinical study, with no requirement for ethical approval.

## Consent

Written informed consent was obtained from the patient for publication of this case report and accompanying images. A copy of the written consent is available for review by the Editor-in-Chief of this journal on request.

## Author contribution

Dr Bianca Kwan: Investigation, Writing – original draft, Writing – Review and Editing, Visualisation.

Dr Jason Wong: Conceptualization, Writing – Review and Editing, Supervision.

## Registration of research studies

Not applicable.

## Guarantor

Dr Bianca Kwan.

Department of Surgery, Redcliffe Hospital, Anzac Avenue, Redcliffe, Queensland, Australia.

Phone: +61 7 3883 7777.

Email: bianca.kwan@health.qld.gov.au.

## Provenance and peer review

Not commissioned, externally peer-reviewed.
